# Clinical and sociodemographic determinants of disease-specific health-related quality of life in long-term breast cancer survivors

**DOI:** 10.1007/s00432-022-04204-w

**Published:** 2022-07-25

**Authors:** Daniela Doege, Melissa S. Y. Thong, Lena Koch-Gallenkamp, Heike Bertram, Andrea Eberle, Bernd Holleczek, Alice Nennecke, Ron Pritzkuleit, Annika Waldmann, Sylke R. Zeissig, Hermann Brenner, Volker Arndt

**Affiliations:** 1 Unit of Cancer Survivorship, Division of Clinical Epidemiology and Aging Research, German Cancer Research Center (DKFZ), PO Box 101949, 69009 Heidelberg, Germany; 2 Division of Clinical Epidemiology and Aging Research, German Cancer Research Center (DKFZ), Heidelberg, Germany; 3 Cancer Registry of North Rhine-Westphalia, Bochum, Germany; 4 Bremen Cancer Registry, Leibniz Institute for Prevention Research and Epidemiology-BIPS, Bremen, Germany; 5 Saarland Cancer Registry, Saarbrücken, Germany; 6 Hamburg Cancer Registry, Hamburg, Germany; 7 Cancer Registry of Schleswig-Holstein, Lübeck, Germany; 8 Institute of Social Medicine and Epidemiology, University Lübeck, Lübeck, Germany; 9 Cancer Registry of Rhineland-Palatinate, Mainz, Germany; 10 Institute of Clinical Epidemiology and Biometry (ICE-B), Julius Maximilian University of Würzburg, Würzburg, Germany; 11 Division of Preventive Oncology, German Cancer Research Center (DKFZ), Heidelberg, Germany; 12 German Cancer Consortium (DKTK), German Cancer Research Center (DKFZ), Heidelberg, Germany

**Keywords:** Quality of life, Breast cancer survivors, Disease-specific issues, Body image, Sexuality, Hair loss, Adverse effects

## Abstract

**Purpose:**

It is important to monitor disease-specific health-related quality of life (HRQoL) in breast cancer (BC) survivors to identify potential unmet supportive care needs. However, previous studies were characterized by small samples of mostly short-term survivors and were limited to certain age ranges, stages and/or treatments.

**Methods:**

We used data from 3045 long-term BC survivors (5–15 years post-diagnosis) recruited in a German multi-regional population-based study. We assessed disease-specific HRQoL with the EORTC QLQ-BR23, scoring from 0 to 100. Differences in functioning and symptoms according to age at survey, self-reported treatments, stage, and disease status (disease-free vs. active disease) were assessed with multiple regression. Active disease was defined as any self-report of recurrence, metastasis or second primary cancer after the index cancer.

**Results:**

Older BC survivors reported a higher body image and a better future perspective, but lower sexual functioning. Survivors aged 30–49 years who had breast-conserving therapy or mastectomy with breast reconstruction reported a better body image compared to those who had mastectomy only. We also found differences in symptoms according to treatments in some age groups. Stage at diagnosis was not associated with HRQoL overall and in most age subgroups. Disease-free BC survivors aged 30–79 years reported a better future perspective and less systemic therapy side effects than those with active disease.

**Conclusion:**

Several treatment-associated symptoms and functioning detriments were found 5–15 years after diagnosis. The results emphasize the need of a comprehensive, individualized survivorship care, recognizing differential needs of long-term BC survivors according to age, treatment modalities, and disease status.

**Supplementary Information:**

The online version contains supplementary material available at 10.1007/s00432-022-04204-w.

## Background

Although overall health-related quality of life (HRQoL) in disease-free long-term breast cancer (BC) survivors (≥ 5 years post-diagnosis) has been reported to be comparable to that of the general population, specific detriments in functioning, more problems with insomnia and dyspnoea, fatigue, and greater financial difficulties still exist (Doege et al. 2019). Besides generic aspects of HRQoL, disease- and treatment-specific issues like e.g. lymphedema, shoulder dysfunction, or neuropathy, can persist even in long-term BC survivors. In an Australian study, more than 60% of BC survivors 6 years after diagnosis still reported at least one ongoing adverse effect (Schmitz et al. 2012). In addition to physical complaints, long-term effects after breast cancer often also involve psychological and emotional issues, like negative body image, fear, distress, and frustration (Taghian et al. 2014). It is important to monitor disease-specific issues and to identify potential supportive care needs of BC survivors, as untreated problems may not dissipate over time and rehabilitation is underutilized in this group (Stubblefield 2017).

Disease-specific HRQoL varies according to treatment (American Cancer Society 2019). Arm and breast symptoms (e.g. lymphedema, pain) are among the most frequent physical long-term consequences for BC survivors. Risk factors include axillary lymph node dissection, mastectomy, chemo- and radiotherapy (Stubblefield 2017; Taghian et al. 2014), age < 70 years at diagnosis and comorbidity (Engel et al. 2003). Symptoms tend to improve little over time, suggesting that they often remain untreated or have been treated unsuccessfully (Engel et al. 2003). A Danish prospective study (*N* = 70) found that even 12 years after surgery, 53% of the included long-term BC survivors still reported pain in the resected breast region and 21% reported arm symptoms, which was confirmed by objective measures (Hauerslev et al. 2020). Higher intensity of pain is associated with lower HRQoL and less return to work (Hamood et al. 2018).

Regarding body image, a recent systematic review found that BC survivors (1 month to 18 years post-treatment) who previously underwent breast-conserving therapy or mastectomy with breast reconstruction reported better body image and physical functioning compared to those after mastectomy without breast reconstruction, while HRQoL in other domains was comparable (Zehra et al. 2020). However, breast reconstruction after mastectomy does not necessarily result in a better body image (Kornblith and Ligibel 2003). The advantage of breast-conserving therapy over mastectomy with regards to sexual functioning is not clear (Kornblith and Ligibel 2003).

Hair disorders can be a further problem in BC patients and survivors. It is widely documented that chemotherapy often leads to (mostly) temporary hair loss and change in shade or texture, which may cause high psychological distress and detriments in HRQoL (Freites-Martinez et al. 2019a). BC survivors who underwent taxane-based chemotherapy have reported permanent hair loss after end of treatment (Freites-Martinez et al. 2019b). Endocrine therapy for BC is also associated with hair thinning or partial hair loss (Freites-Martinez et al. 2019a).

Disease-specific issues after BC have often been studied in BC patients during/ after treatment or in short-term BC survivors (< 5 years post-diagnosis), and only few studies focused on long-term BC survivors. Further, previous studies are often based on small samples, low stages, or limited to certain age ranges or certain treatments. This study aims to use a large, population-based cohort of long-term BC survivors (5–15 years post-diagnosis) with a broad age range and to include all stages and treatments to allow for comparison. Different subgroups of long-term BC survivors might have different care and support needs and thus the study may identify these needs more specifically.

## Methods

### Sample

The sample of 3045 BC survivors (mean 65.3 years) was recruited in a German multi-regional population-based study (CAESAR+). Details of the study have been reported elsewhere (Arndt et al. 2017; Doege et al. 2019). In short, the CAESAR+ study included long-term breast, colorectal and prostate cancer survivors diagnosed between 1994 and 2004, and reported to one of six participating German cancer registries (Bremen, Hamburg, North Rhine–Westphalia, Rhineland–Palatinate, Saarland, and Schleswig–Holstein). Inclusion criteria were age at diagnosis 20–75 years and a histological confirmation of the cancer. Participants answered postal questionnaires between March 2008 and May 2011. Non-respondents received up to two reminder letters and a telephone contact. Out of 6553 contacted BC survivors, 3045 completed the full-length questionnaire (response rate: 46.5%) and were included in the present analysis. The study was approved by the responsible institutional ethics committees. Written informed consent was obtained from each participant.

### Measurements

Disease-specific aspects of HRQoL were assessed by the European Organization for Research and Treatment of Cancer Breast Cancer–Specific Quality of Life Questionnaire (EORTC QLQ-BR23). The 23-item questionnaire consists of four functioning items/scales (body image, future perspective, sexual functioning, and sexual enjoyment) and four symptom items/scales (arm symptoms, breast symptoms, systemic therapy side effects, and upset by hair loss). The conditional questions on sexual enjoyment and upset about hair loss are only completed if the participant is sexually active or has hair loss, respectively. Answers range from 1 (not at all) to 4 (very much) for all items. Linear transformation of raw scores to scales of 0–100 was performed according to the EORTC scoring manual (Fayers et al. 2001). High scores on the functioning scales indicate better functioning. On the symptom scales, a high score represents a greater burden.

The participating cancer registries provided additional clinical information such as year of diagnosis and cancer stage. Information on treatment and on recurrence, metastasis or new cancer since initial diagnosis were assessed via self-report. “Active disease” was defined as either any self-report of recurrence, metastasis or second primary cancer after the study cancer.

### Statistical analysis

Differences in BR23 items/scales were assessed with multiple regression, overall and stratified by age at survey, education, and clinical variables. We categorized age at survey for stratification as follows: 30–49, 50–59, 60–69, 70–79, and 80–89 years. Education was categorized as ≤ 9 years, 10–11 years, ≥ 12 years (according to the German schooling system). Stage at diagnosis was classified as stage I, II, III, and IV (according to UICC TNM 7th edition). Type of surgery was stratified as “breast-conserving”, “mastectomy with reconstruction” (irrespective whether immediate or delayed), and “mastectomy without reconstruction”, excluding BC survivors without surgery or unusual combinations of surgeries. Further self-reported treatment modalities (chemotherapy, endocrine therapy, radiotherapy), lymph node dissection, as well as active disease, were each dichotomized as yes/no. For the stratification according to active disease, we excluded BC survivors with stage IV at diagnosis.

All analyses were additionally adjusted for age at survey (categorized as 30–44, 45–49, 50–54, 55–59, 60–64, 65–69, 70–74, 75–79, 80–84, and 85–89 years), education (categorized as ≤ 9 years, 10–11 years, ≥ 12 years), time since diagnosis (categorized as 5–9 and ≥ 10 years), stage (I–IV), active disease (yes/no), type of surgery (“no surgery”, “breast-conserving”, “breast-conserving and reconstruction”, “mastectomy and reconstruction”, and “mastectomy and no reconstruction”), chemotherapy (yes/no), endocrine therapy (yes/no), radiotherapy (yes/no), and lymph node dissection (yes/no), where appropriate.

We employed multiple imputation, based on the Markov Chain Monte Carlo method with 25 repetitions, to reduce possible bias due to missing values (in general less than 10%). Conditional items were excluded from the imputation. All analyses were conducted with SAS (version 9.4 for Windows; SAS Institute Inc., Cary, NC). A *p* value < 0.05 (two-sided) was considered statistically significant. The *p* values were not adjusted for multiple testing, referring to the individual tests rather than a global test for differences.

## Results

The non-responder analysis has been reported elsewhere (Doege et al. 2019). In short, respondents were slightly younger at diagnosis (mean 57.1 vs. 57.6 years) and at the time of the survey (mean 65.3 vs. 66.2 years), they had a shorter time since diagnosis (mean 8.2 vs. 8.6 years), and they were less likely to have stage IV disease (1.2 vs. 2.0%). However, the distribution of local and regional tumor extension and of stage I–III did not differ significantly between respondents and non-respondents.

Table 1 shows the sociodemographic and clinical characteristics of the respondents. At time of recruitment, participants were on average 65 years old. Fifty-four percent of the participants reported nine or fewer years of education, 17% reported 12 or more years of education. Almost 22% were still employed full or part time. Seventy-one percent of the survivors were partnered and 85% had children. Most participants were still living in their own household. Heart failure (9%), diabetes (11%), and depression (23% ever) were the most frequent comorbidities. Regarding clinical factors, 90% of BC survivors had stage I or II disease. Seventy-seven percent were 5–9 years post-diagnosis, the rest 10–16 years. The majority (62%) had undergone breast-conserving therapy, 7% reported mastectomy with breast reconstruction and 24% had undergone mastectomy without breast reconstruction. Ninety-five percent of survivors reported having had lymph node dissection, 84% had radiotherapy, 60% had chemotherapy, and 50% had endocrine therapy. Fourteen percent of survivors were classified as having active disease.

**Table 1:** Sample characteristics

		*n*	%
Total		3045	100.0
Mean age (SD)		65.3	(9.6)
Age at survey (0.0% missings^a^)	30–49 years	219	7.2
	50–59 years	603	19.8
	60–69 years	1098	36.1
	70–79 years	973	32.0
	80–89 years	152	5.0
Education ﻿(1.3% missings)	≤ 9 years	1653	54.3
	10 years	858	28.2
	≥ 12 years	534	17.5
Employment (1.9% missings)	Full-time	230	7.6
	Part-time	437	14.4
	Unemployed	47	1.6
	Housewife	701	23.0
	(Early) Retirement	1521	50.0
	Other	97	3.2
	Multiple answers	12	0.4
Have a partner (13.9% missings)		2169	71.2
Living with a partner (3.0% missings)		2038	66.9
Have children (0.4% missings)		2585	84.9
Living situation (0.3% missings)	Own household	3013	99.0
	With others	17	0.6
	Nursing home	15	0.5
Comorbidities (self-report, 2.4–4.3% missings)	Stroke	78	2.6
	Myocardial infarction	58	1.9
	Heart failure	286	9.4
	Diabetes mellitus	327	10.8
	Depression (ever)	703	23.1
Stage (UICC, considering MX as M0, 7.7% missings)	I	1351	44.4
	II	1407	46.2
	III	251	8.3
	IV	35	1.2
Time since diagnosis (0.2% missings)	5–9 years	2341	76.9
	10–16 years	704	23.1
Type of surgery (self-report, 1.8% missings)	No surgery	153	5.0
	Breast-conserving and no reconstruction	1876	61.6
	Breast-conserving and reconstruction	63	2.1
	Mastectomy and reconstruction	225	7.4
	Mastectomy and no reconstruction	728	23.9
Further treatment (self-report, 2.6–9.9% missings)	Lymph node dissection	2877	94.5
	Radiotherapy	2555	83.9
	Chemotherapy	1831	60.1
	Endocrine therapy	1531	50.3
Active disease^b^ (1.7% missings)		398	13.0

^a^Percentage of missing values per variable before multiple imputation

^b^Any self-report of recurrence, metastasis or second primary cancer after the study cancer, proportion refers to *N* = 3010 BC survivors stage I–III. All frequencies and percentages are based on 25 imputations of missing values

### Functioning and symptoms according to age and education

**Fig. 1:**
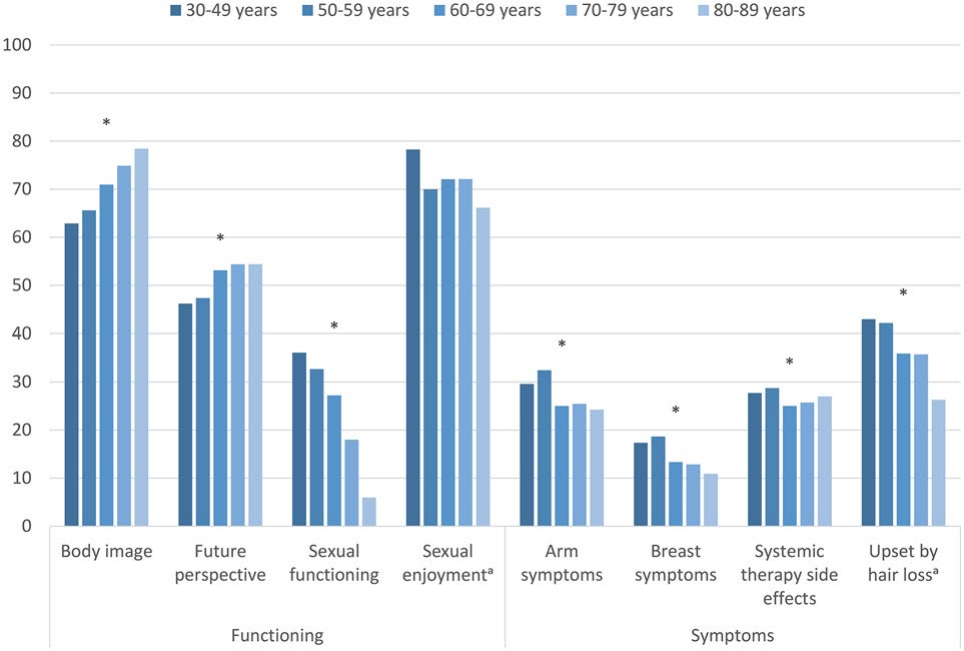
EORTC BR23 functioning and symptom scores and items of long-term breast cancer survivors, stratified by age, adjusted for education, stage, type of surgery, chemotherapy, endocrine therapy, radiotherapy, lymph node dissection, time since diagnosis, and active disease. Footnotes: ^a^conditional items (no imputation, *N* = 934 participants for sexual enjoyment and *N* = 1442 participants for upset by hair loss). All further results are based on 25 imputations of missing values. Asterisks (*) mark statistically significant differences (*p* < 0.05) in global comparison. High scores on body image, future perspective, sexual functioning, and sexual enjoyment indicate better functioning. High scores on arm symptoms, breast symptoms, systemic therapy side effects, and upset by hair loss indicate greater burden

Figure 1 shows the mean scores for all EORTC BR23 functioning and symptom scales, stratified by age groups. Better body image scores were reported in higher age groups (Δ_max_ 15.6 scale points between groups), as well as a better future perspective (Δ_max_ 8.2) whereas sexual functioning scores was higher for younger BC survivors (Δ_max_ 30.1). Sexual enjoyment showed no statistically significant association with age. Upset by hair loss was higher at younger ages (Δ_max_ 16.7). For arm and breast symptoms and for systemic therapy side effects, the age group 50–59 years reported most issues (Δ_max_ 8.2, 7.8, and 3.7, respectively). However, these symptom scores were generally low.

Participants with 9 or less years of education reported significantly lower functioning on all scales, and more arm and breast symptoms, as well as systemic therapy side effects, compared to participants with 10 or more years of education (Δ_max_ between 4.1 and 11.3, supplementary material, Table S1). When further stratifying by age, the overall differences in functioning according to education were only found in some of the age subgroups, and the pattern was inconsistent (supplementary material, Fig. S1). Regarding symptoms, lower education was associated with more arm symptoms and systemic therapy side effects in the age groups below 80 years, and with more breast symptoms in the age groups below 70 years (supplementary material, Fig. S2).

### Functioning according to type of surgery

**Fig. 2:**
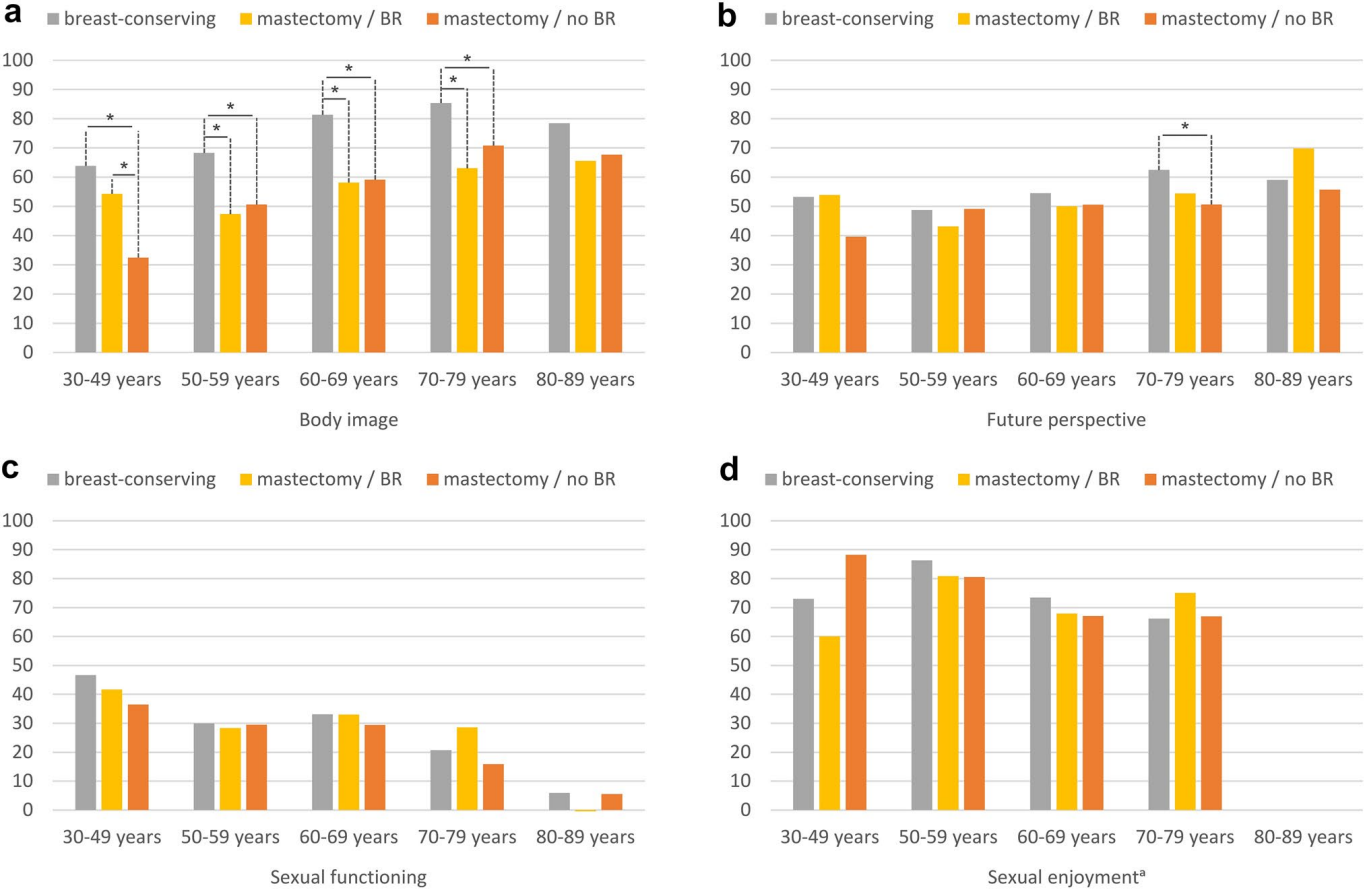
EORTC BR23 functioning scores and items of long-term breast cancer survivors, stratified by age and type of surgery, adjusted for education, stage, chemotherapy, endocrine therapy, radiotherapy, lymph node dissection, time since diagnosis, and active disease. : ^a^conditional item (no imputation, *N* = 888 participants). All further results are based on 25 imputations of missing values. The analysis is based on *N* = 2829 participants (exclusion of 216 BC survivors who either reported no surgery or an unusual combination of surgeries). High scores indicate better functioning. Asterisks (*) mark statistically significant differences in pairwise comparison (*p* < 0.05). The spans of the lines indicate which subgroups differ significantly in pairwise comparison, e.g. if the line spans three columns, it indicates a significant difference between breast-conserving therapy and mastectomy without reconstruction. Example: participants aged 30–49 years who had breast-conserving therapy, as well as participants who had mastectomy with reconstruction (mastectomy/BR), report a better body image than participants who had mastectomy without reconstruction (mastectomy/no BR), whereas women with breast-conserving therapy and women who had mastectomy with reconstruction are not significantly different

Regarding type of surgery, overall statistically significant differences were found for body image, future perspective, and sexual functioning (Table 2). Differences were largest for body image, whereby BC survivors after breast-conserving treatment reported a better body image than those who had mastectomy with breast reconstruction (Δ 22.0) and those who had mastectomy alone (Δ 17.4). When additionally stratifying by age (Fig. 2a), for survivors aged between 30 and 49 years, no statistically significant difference in body image were found between breast-conserving operation and mastectomy with breast reconstruction, and both groups reported better body image than survivors who had mastectomy alone (Δ_max_ 31.4). In the age groups between 50 and 79 years, as in the overall sample, BC survivors reported better body image after having had breast-conserving operation compared to mastectomy, irrespective of whether the mastectomy was followed by breast reconstruction or not (Δ_max_ 23.2). The same pattern was found in survivors aged 80–89 years, but the differences were not statistically significant.

**Table 2:** Mean EORTC BR23 functioning and symptom scores and items of long-term breast cancer survivors, stratified by treatment, stage, and active disease. Adjusted for age, education, stage, type of surgery, chemotherapy, endocrine therapy, radiotherapy, lymph node dissection, time since diagnosis, and active disease, where appropriate

Type of surgery	Breast-conserving	Mastectomy/BR^c^	Mastectomy/no BR^c^		*p*
Body image	80.7	58.7	63.3		** < 0.0001**
Future perspective	56.5	49.9	51.3		**0.0130**
Sexual functioning	27.1	30.8	21.4		** < .0001**
Sexual enjoyment^a^	75.1	70.3	70.1		0.2368
**Lymph node dissection**	**No**	**Yes**			***p***
Arm symptoms	22.6	31.4			**0.0001**
Breast symptoms	11.8	17.1			**0.0029**
**Radiotherapy**	**No**	**Yes**			***p***
Arm symptoms	25.5	28.5			0.1027
Breast symptoms	12.8	16.1			**0.0193**
**Chemotherapy**	**No**	**Yes**			***p***
Systemic therapy side effects	26.0	26.7			0.3906
Upset by hair loss^a^	34.2	40.3			**0.0079**
**Endocrine therapy**	**No**	**Yes**			***p***
Systemic therapy side effects	25.8	27.0			0.1044
Upset by hair loss^a^	36.3	38.1			0.4041
**Stage at diagnosis**	**Stage I**	**Stage II**	**Stage III**	**Stage IV**	***p***
Body image	70.6	70.7	70.7	70.8	0.9750
Future perspective	51.4	52.3	52.3	50.8	0.8969
Systemic therapy side effects	27.0	26.5	27.7	24.3	0.6426
Upset by hair loss^a^	36.0	40.2	41.1	31.6	0.1943
**Active disease**^b^	**No**	**Yes**			***p***
Body image	73.8	67.8			**0.0001**
Future perspective	59.1	45.5			** < 0.0001**
Systemic therapy side effects	23.9	30.1			** < 0.0001**
Upset by hair loss^a^	35.9	42.3			**0.0266**

Bold *p* values mark statistically significant differences (*p* <0 .05) in global comparison. The analysis on type of surgery is based on *N* = 2829 participants (exclusion of 216 BC survivors who either reported no surgery or an unusual combination of surgeries). The analysis on active disease is based on 3010 participants (exclusion of 35 BC survivors with stage IV disease)

breast reconstruction, “Active disease”: any self-report of recurrence, metastasis or second primary cancer after the study cancer

^a^Conditional items (no imputation, *N* = 934 participants for sexual enjoyment and *N* = 1442 participants for upset by hair loss). All further results are based on 25 imputations of missing values.

^b^Defined as any self-report of recurrence, metastasis or second primary cancer after the study cancer

^c^Breast reconstruction

BC survivors who received breast-conserving treatment also reported a more positive future perspective than those who had undergone mastectomy without breast reconstruction (Δ 5.2), while future perspective of those who underwent mastectomy with breast reconstruction did not differ statistically significant from that of any other group. Looking at age-specific differences (Fig. 2b), the same result was found for BC survivors aged 70–79 years (Δ 11.8), while in the other age groups, no statistically significant differences were found.

BC survivors with breast-conserving surgery and those with mastectomy with breast reconstruction indicated better sexual functioning than those with mastectomy alone (Table 2, Δ_max_ 9.5). However, in age-specific comparisons, no statistically significant differences were found (Fig. 2c). For sexual enjoyment, there were no statistically significant differences according to type of surgery (Table 2, Fig. 2d).

### Arm and breast symptoms

**Fig. 3:**
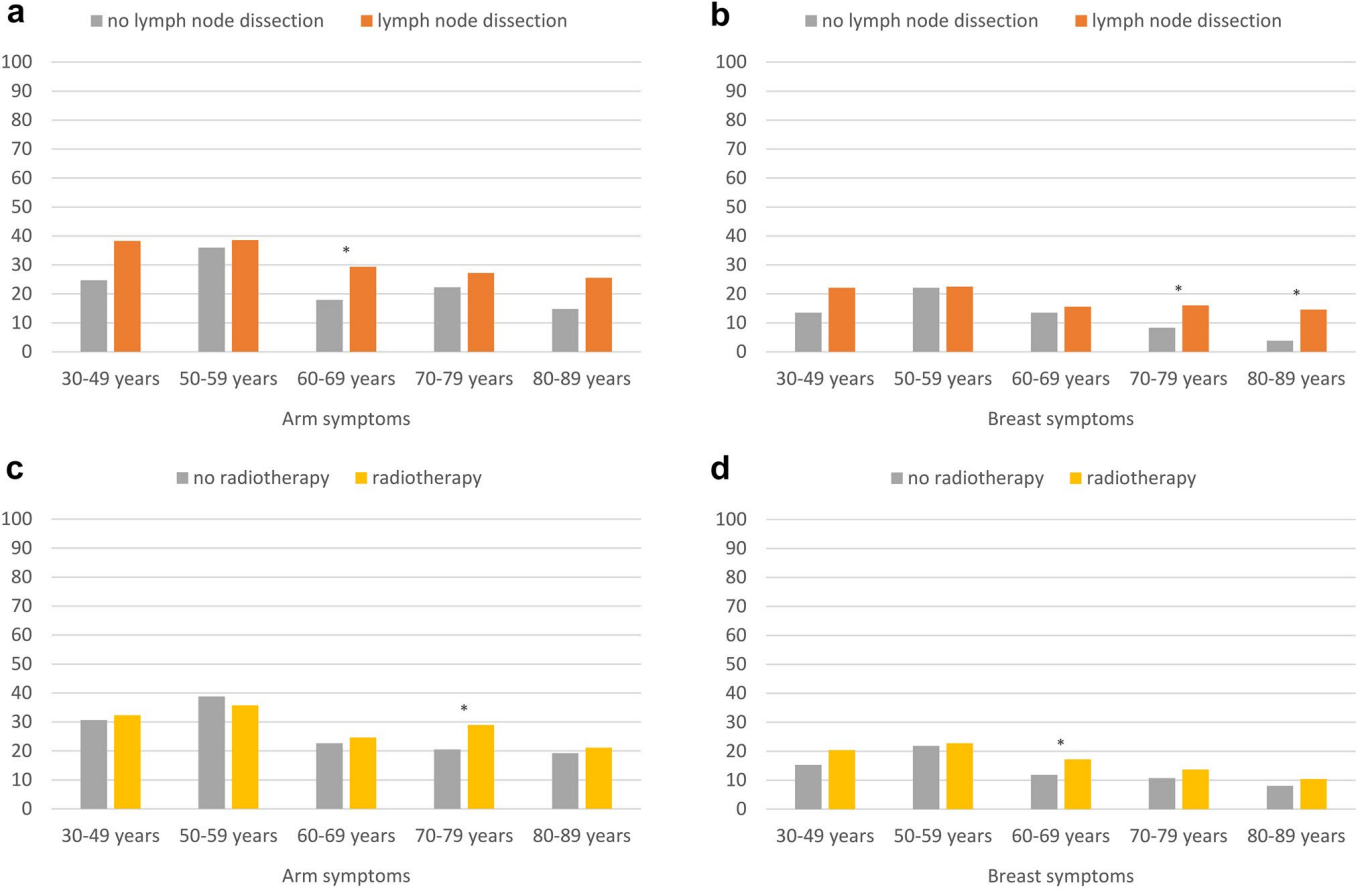
Arm and breast symptoms (EORTC BR23) of long-term breast cancer survivors, stratified by age and treatment, adjusted for education, stage, type of surgery, chemotherapy, endocrine therapy, radiotherapy, lymph node dissection, time since diagnosis, and active disease, where appropriate. Footnotes: All results are based on 25 imputations of missing values. High scores indicate greater burden. Asterisks (*) mark statistically significant differences (*p* < 0.05)

BC survivors who underwent lymph node dissection, compared to those who did not undergo lymph node dissection, reported significantly more persisting arm (Δ 8.8), and breast symptoms (Δ 5.3) (Table 2). When further stratifying by age, differences were only found in some age groups (Fig. 3a, b). BC survivors who underwent lymph node dissection, compared to those without lymph node dissection, reported more arm symptoms in the age group 60–69 years (Δ 11.4) and more breast symptoms in the age groups 70–89 years (Δ_max_ 10.7).

BC survivors who received radiotherapy, overall reported more breast symptoms than survivors not treated with radiotherapy (Δ 3.4), while there was no statistically significant difference for arm symptoms (Table 2). Looking at age-specific differences, only those in the age group 60–69 years reported more breast symptoms (Δ 8.4) and those in the 70–79 years age group reported more arm symptoms after having received radiotherapy, compared to no radiotherapy (Δ 5.3, Fig. 3c, d). No statistically significant differences according to radiotherapy were found in the other age groups.

### Systemic therapy side effects and upset by hair loss

**Fig. 4:**
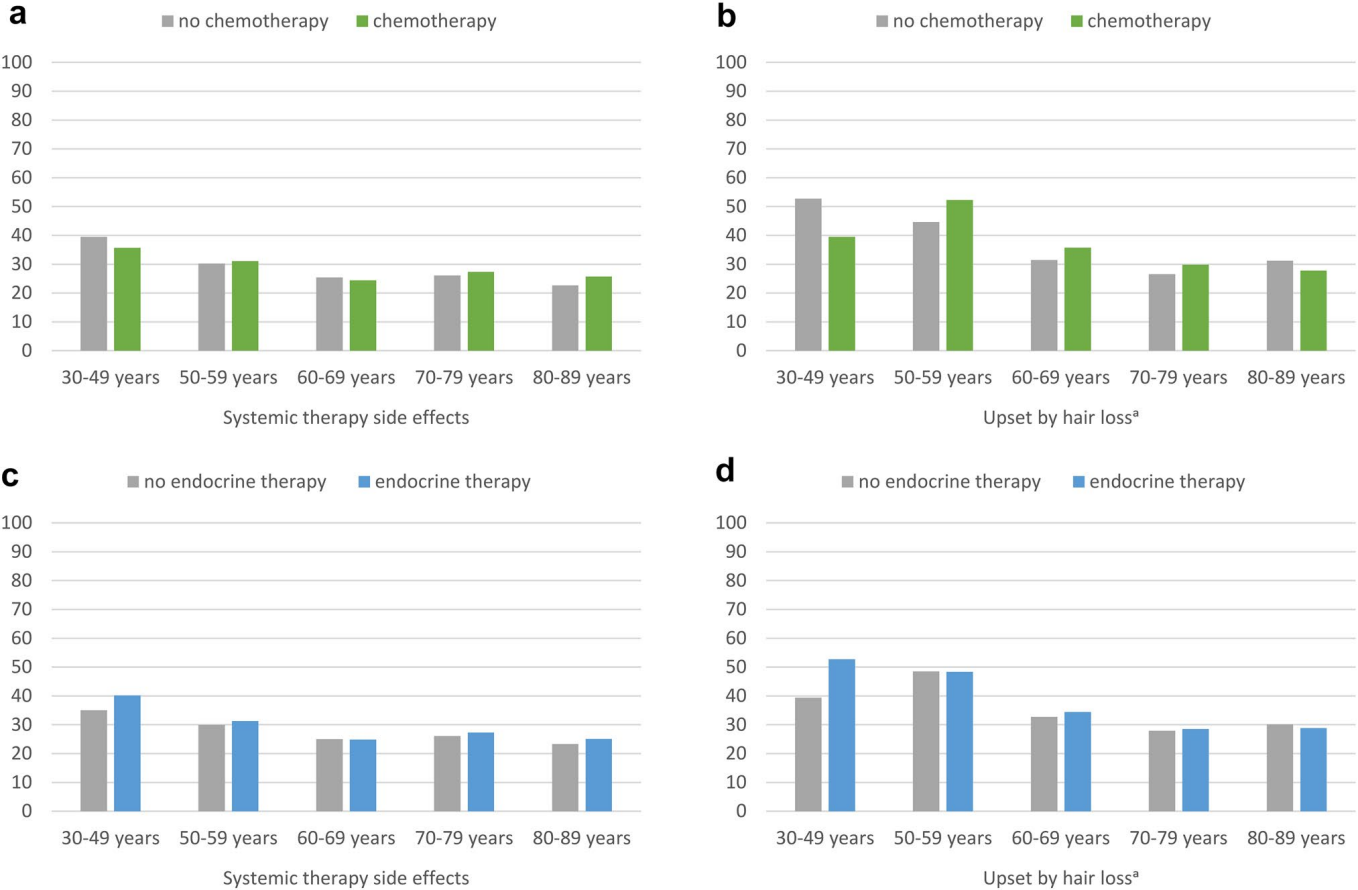
Systemic therapy side effects and upset by hair loss (EORTC BR23) of long-term breast cancer survivors, stratified by age and treatment, adjusted for education, stage, type of surgery, chemotherapy, endocrine therapy, radiotherapy, lymph node dissection, time since diagnosis, and active disease, where appropriate. Footnotes: ^a^conditional item (no imputation, *N* = 1442 participants). All further results are based on 25 imputations of missing values. High scores indicate greater burden. Asterisks (*) mark statistically significant differences (*p* < 0.05)

We further compared whether chemotherapy or endocrine therapy were associated with systemic therapy side effects or with upset by hair loss. Overall, upset by hair loss was more frequent in BC survivors who had undergone chemotherapy, compared to no chemotherapy (Δ 6.1, Table 2). However, when looking at age-specific patterns, no statistically significant differences were found according to chemo- or endocrine therapy (Fig. 4a–d).

### Functioning and symptoms according to stage at diagnosis

**Fig. 5:**
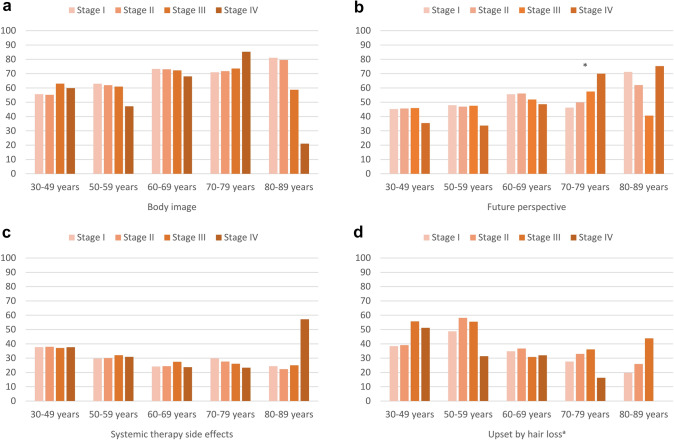
Selected EORTC BR23 functioning and symptom scores and items of long-term breast cancer survivors, stratified by age and stage, adjusted for education, type of surgery, chemotherapy, endocrine therapy, radiotherapy, lymph node dissection, active disease, and time since diagnosis. Footnotes: ^a^conditional item (no imputation, *N* = 1442 participants). All further results are based on 25 imputations of missing values. High scores on body image and future perspective indicate better functioning, high scores systemic therapy side effects and upset by hair loss indicate greater burden. Asterisks (*) mark statistically significant differences (*p* < 0.05)

Overall, we did not find any statistically significant differences in functioning or symptoms of BC survivors according to their stage at diagnosis, after adjusting for all other factors (Table 2). In age-specific comparisons, there was only one globally statistically significant difference, namely for future perspective in age group 70–79 years (Δ_max_ 23.7, Fig. 5b). In this age group, BC survivors with stage IV reported significantly better future perspective than BC survivors with stage I/II (*p* = 0.01/ 0.03, data not shown).

### Functioning and symptoms according to disease status

**Fig. 6:**
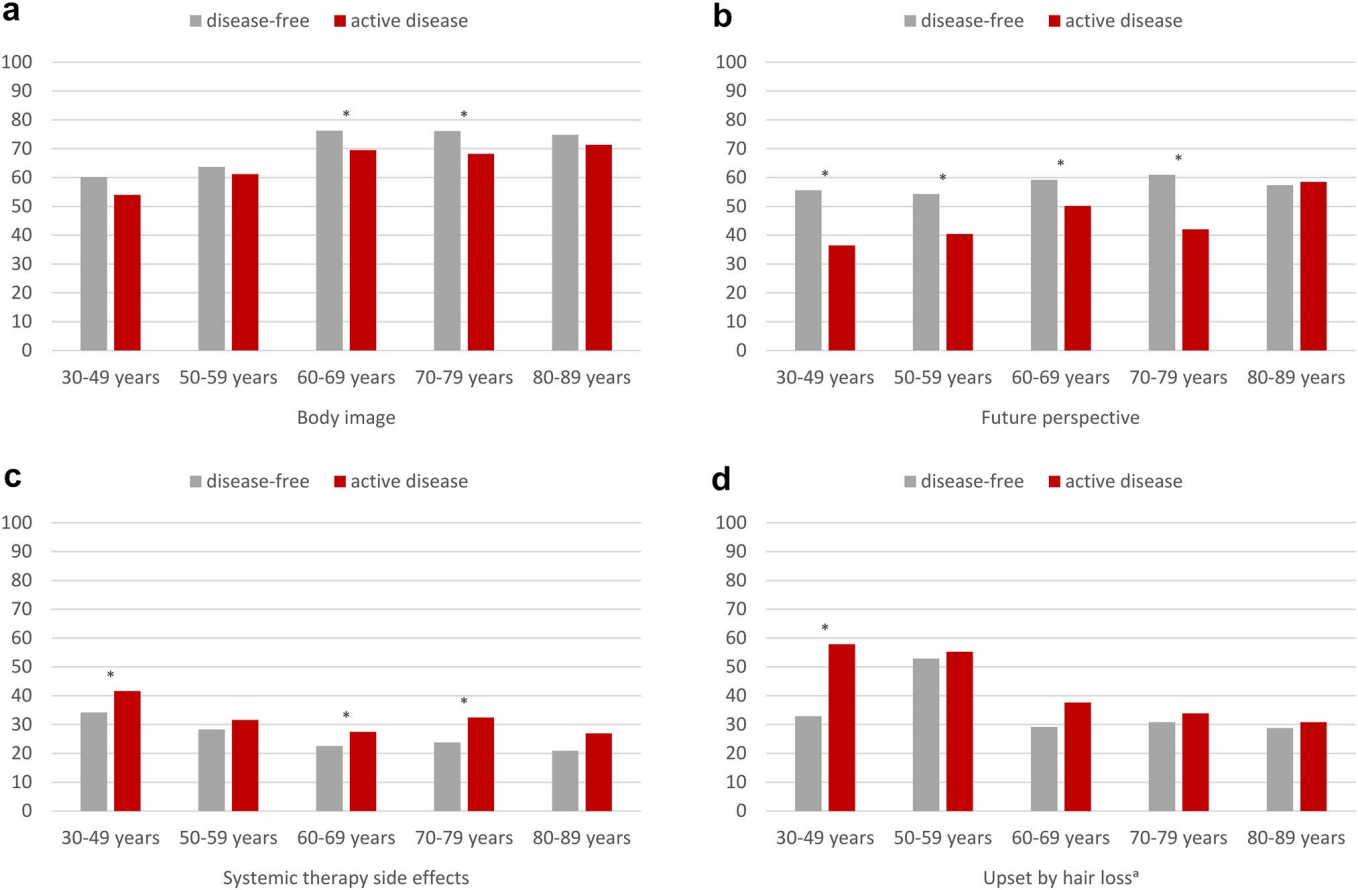
Selected EORTC BR23 functioning and symptom scores and items of long-term breast cancer survivors, stratified by age and active disease, adjusted for education, stage, type of surgery, chemotherapy, endocrine therapy, radiotherapy, lymph node dissection, and time since diagnosis. Footnotes: “Active disease”: Any self-report of recurrence, metastasis or second primary cancer after the study cancer. The analysis is based on 3010 participants (exclusion of 35 BC survivors with stage IV disease). ^a^Conditional item (no imputation, *N* = 1419 participants). All further results are based on 25 imputations of missing values. High scores on body image and future perspective indicate better functioning, high scores systemic therapy side effects and upset by hair loss indicate greater burden. Asterisks (*) mark statistically significant differences (*p* < 0.05)

Overall, BC survivors stage I–III with active disease reported worse body image (Δ 6.0) and future perspective (Δ 13.6), and more systemic therapy side effects (Δ 6.2) and upset by hair loss (Δ 6.3). Further stratifying by age, participants with active disease, compared to disease-free survivors, in the age groups 60–79 years reported a worse body image (Δ_max_ 7.9, Fig. 6a) and more systemic therapy side effects (Δ_max_ 8.6, Fig. 6c). In all age groups below 80 years, BC survivors with active disease reported worse future perspective (Δ_max_ 19.1, Fig. 6b). BC survivors with active disease aged 30–49 years also reported more systemic therapy side effects (Δ 7.4, Fig. 6c) and they were more upset by hair loss than same-aged disease-free BC survivors (Δ 25.0, Fig. 6d).

## Discussion

This study analyzed disease-specific aspects of HRQoL in a large population-based cohort of long-term BC survivors (5–15 years post-diagnosis). In general, we found that age, education, and, to some lesser extent, treatment- and disease-related variables were associated with disease-specific HRQoL. BC survivors with lower educational levels reported more symptoms than those with higher education, especially at younger ages. It has been reported before that socio-economic deprivation of BC survivors is associated with lower quality of life, which might be explained by lower uptake of follow-up care and more stress due to lower socio-economic status (Dialla et al. 2015).

BC survivors reported poorer body image after mastectomy, in which breast reconstruction only showed a beneficial effect for body image in the age group 30–49 years, but not for women aged 50 years and above. This is in line with a systematic review finding that breast reconstruction is more relevant for body image in younger BC survivors (Paterson et al. 2016). According to another systematic review, previous studies on the effect of breast reconstruction in general found discrepant results for different aspects of body image (Fang et al. 2013). Previous studies also found breast reconstruction to be less beneficial for BC survivors’ body image than expected (Rowland et al. 2000; Janz et al. 2005). The process of breast reconstruction usually takes more time and might be more stressful than mastectomy alone (Rowland et al. 2000), and coping with the cosmetic result could be more difficult than coping with mastectomy (Janz et al. 2005). Some BC survivors might be disappointed with the outcome or with the fact that breast-conserving treatment was not possible (Janz et al. 2005; Rowland et al. 2000).

Despite the long time since diagnosis, BC survivors in age groups 60 years and above reported more breast and arm symptoms after lymph node dissection and radiotherapy. However, results were mixed and in some age and treatment subgroups there was no difference in arm and breast symptoms according to treatment. A previous prospective study showed that an effect of axilla surgery on arm problems was dependent on the number of harvested lymph nodes (Engel et al. 2003), and in our study, this information was not available. Overall, breast and arm symptoms decreased with increasing age and were more a problem of those BC survivors still at working age, which is in line with previous studies (Engel et al. 2003; Macdonald et al. 2005).

Body image and future perspective were positively associated with age, and upset by hair loss was less of a problem in older survivors. As they age, BC survivors might shift values, have a more relaxed view on themselves and the future, and are more at peace with their physical appearance. A systematic review showed that body image was lower in younger BC survivors, but without a difference to same-aged controls (Davis et al. 2020).

The BC survivors in this study had long completed their therapy and we did not find any long-term associations between chemo- or endocrine therapy and systemic therapy side effects. Further, only a few BC survivors reported (permanent) hair loss, leading to a small sample size who answered the item “upset by hair loss”. Nevertheless, we found that younger survivors were more likely to be upset by hair loss. However, no statistically significant associations of being upset by hair loss were found according to endocrine therapy, and associations with chemotherapy were only visible in the overall group, but not in age-stratified analyses. When receiving chemotherapy, affected women might know that hair loss is mostly temporary, so the differences are not reflected in upset about hair loss. Hair loss might also be a reason to terminate endocrine therapy early (Karatas et al. 2016), which was not controlled for in our study. We also did not explicitly assess hair thinning or other qualitative changes of (regrown) hair. According to a Japanese study, 59% of BC survivors 4–5 years post-diagnosis reported a scalp hair recovery of > 80%, 35% reported only 40–70% recovery, and in general, the hair was often reported to regrow thinner, curlier or greyer (Watanabe et al. 2019).

In our study, sexual functioning, which in the questionnaire relates to sexual interest and the frequency of being sexually active, was lower at older age. The decrease in sexual activity with age, irrespective of treatment, is in line with previous studies in long-term BC survivors (Soldera et al. 2018; Ganz et al. 2002). However, sexually active women in all age groups reported comparable levels of sexual enjoyment.

There was no clear association between tumor stage at diagnosis and disease-specific HRQoL. We only found one statistically significant difference in the age group 70–79 years, in which BC survivors with stage IV reported a better future perspective than stage I and II. This specific subgroup of elderly survivors with initially poorer prognosis are still alive longer than expected based on their age and stage (mean time since diagnosis in this group: 9.0 years), which could have resulted in more hope and optimism. However, given that the pattern is solely found in one age group and the number of survivors with stage IV is small, it is unlikely that the result reflects a systematic effect.

BC survivors below 80 years with active disease reported a worse future perspective than disease-free BC survivors of the same age. Cancer recurrence is often associated with hopelessness, psychological stress (Brothers and Andersen 2009), and lower quality of life (Arndt et al. 2005). Active disease was also associated with more side effects in certain age groups, which might be due to additional or more intensive or burdensome treatments. Although the analyses were adjusted for treatments (including primary treatment and treatment of recurrence), the intensity of those treatments, like chemotherapy doses or radiation circles were not assessed and thus could not be considered for adjustment. We also did not consider the year when the treatments were undergone, as this information was reported only by a subset of the respondents.

Some further limitations have to be considered when interpreting the study results. There is a risk of healthy survivor bias, as BC survivors who were of higher age and had more health problems might have been less likely to participate in the study. This would result in an underestimation of remaining health problems. We did not compare functioning and symptoms to normative data or a control group as the BR23 is not applicable in women without breast cancer. Further, participants were recruited 5 and more years after diagnosis and the study was cross-sectional. As such, a potential underassessment of the risk for late and long-term sequelae due to a prevalence-incidence bias (Neyman bias (Hill et al. 2003)) has to be considered. Treatments were self-reported by yes-/no-questions and might be biased due to memory effects, although we assume that most participants remembered burdensome treatments like operations or chemotherapy. The timing and intensity of treatments were not considered in this study. Further, some treatments were reported by a vast majority of the sample (e.g. lymph node dissection) which led to very unequal group sizes for comparison. Some treatments and guidelines might have changed since the time when the study cohort was diagnosed, e.g. more modern, less invasive or lower-dosed treatments, newer therapeutic agents or treatment regimens, or a lower rate of lymph node dissection at earlier stages (Fisher et al. 2020). In recent years, shared decision-making has also gained more popularity. Newly diagnosed BC patients might have more possibilities to actively participate and make an informed treatment decision, compared to the BC survivors in our sample, which might result in a better sense of control over potential late-effects.

When analyzing active disease, we did not control for the date of recurrence, metastasis or second cancer due to the overall small number of such events. Differences in functioning and symptoms according to age reflect the cross-sectional comparison of age cohorts rather than the longitudinal development of individuals. Some important information such as cancer stage was not fully reported to the cancer registries in the period when study participants were first diagnosed (7.7% missings). To overcome this, we replaced missing data using multiple imputation and ran sensitivity analyses which showed that the imputed results were similar to those from non-imputed data. Another limitation of the study is that we restricted the subgroup analyses on disease-specific problems to age, education, clinical and treatment-related variables. Future studies might focus more on sociodemographic and psychosocial factors like job situation or social support to identify special support needs and, if needed, starting points for intervention.

Strengths of the study include the large sample size that was recruited in a multi-regional, population-based study. The large sample size allowed us to stratify groups for more in-depth analyses and adjust the results by various clinical and sociodemographic variables, increasing the validity of the results for different subgroups of long-term BC survivors.

In a previous study, we have shown that the disease-free subsample of BC survivors in our study reported a HRQoL that was comparable to that of same-aged population controls (Doege et al. 2019). Similarly, it is heartening that in the current study, BC survivors reported, in general, no potential long-term side effects of self-reported chemotherapy and endocrine therapy. However, for BC survivors in certain age groups, some disease-specific issues were still of concern even long after diagnosis. For example, younger survivors reported a worse body image after mastectomy and more arm and breast symptoms after lymph node dissection and radiotherapy. Therefore, it is important for clinicians and for BC survivors to know that some symptoms and functioning deficits can persist over years and that needs of long-term BC survivors differ according to age, education, treatment, and disease status. BC patients diagnosed in recent years might profit from advances in shared decision-making, enabling them to find their personal balance between risks and benefits of the treatments they undergo, e.g. regarding breast reconstruction. Patients with lower educational levels and potentially lower health literacy might profit from clear and simple explanations regarding treatment options and potential side effects to gain more sense of control. BC survivors should be encouraged to bring potential ongoing physical and psychological problems and concerns to the attention of health care providers. Further research should evaluate if and to what extent a standardized screening of patient-reported outcomes at different stages of the survivorship trajectory might help to identify specific needs and to support vulnerable BC survivors to improve their coping process.

## Supplementary Information

Below is the link to the electronic supplementary material.

Supplementary file1 (DOCX 566 kb)

## Data Availability

The data presented in this study are available on request from the corresponding author.
