# Supplementary material for: Clinical and sociodemographic determinants of disease-specific health-related quality of life in long-term breast cancer survivors
Source: J Cancer Res Clin Oncol. 2022 Jul 25;148(12):3461–73. doi: 10.1007/s00432-022-04204-w (PMC9587098; doi:10.1007/s00432-022-04204-w)
Supplement: Supplementary file 1 — Supplementary file1 (DOCX 566 kb) [file 432_2022_4204_MOESM1_ESM.docx]

Supplementary material for
„Clinical and sociodemographic determinants of disease-specific health-related quality of life in long-term breast cancer survivors” (Doege et al., 2022)

**Table S1:** Mean EORTC BR23 functioning and symptom scores and items of long-term breast cancer survivors, stratified by education. Adjusted for age, stage, type of surgery, chemotherapy, endocrine therapy, radiotherapy, lymph node dissection, time since diagnosis, and active disease.

| **Education** | **≤ 9 years** | **10 years** | **≥ 12 years** | **p** |
| --- | --- | --- | --- | --- |
| Body image | 69.3 | 73.4 | 72.0 | **0.0014** |
| Future perspective | 48.8 | 55.7 | 56.7 | **<.0001** |
| Sexual functioning | 21.6 | 27.3 | 28.0 | **<.0001** |
| Sexual enjoymentᵃ | 67.0 | 72.4 | 74.6 | **0.0023** |
| Arm symptoms | 32.6 | 25.5 | 21.3 | **<.0001** |
| Breast symptoms | 17.7 | 13.2 | 11.5 | **<.0001** |
| Systemic therapy side effects | 28.5 | 25.3 | 22.9 | **<.0001** |
| Upset by hair lossᵃ | 35.3 | 37.5 | 33.7 | 0.4260 |

*Footnotes: ^a^ conditional items (no imputation, N = 934 participants for sexual enjoyment and N = 1442 participants for upset by hair loss). All further results are based on 25 imputations of missing values. Bold p-values mark statistically significant differences (p < .05) in global comparison.*


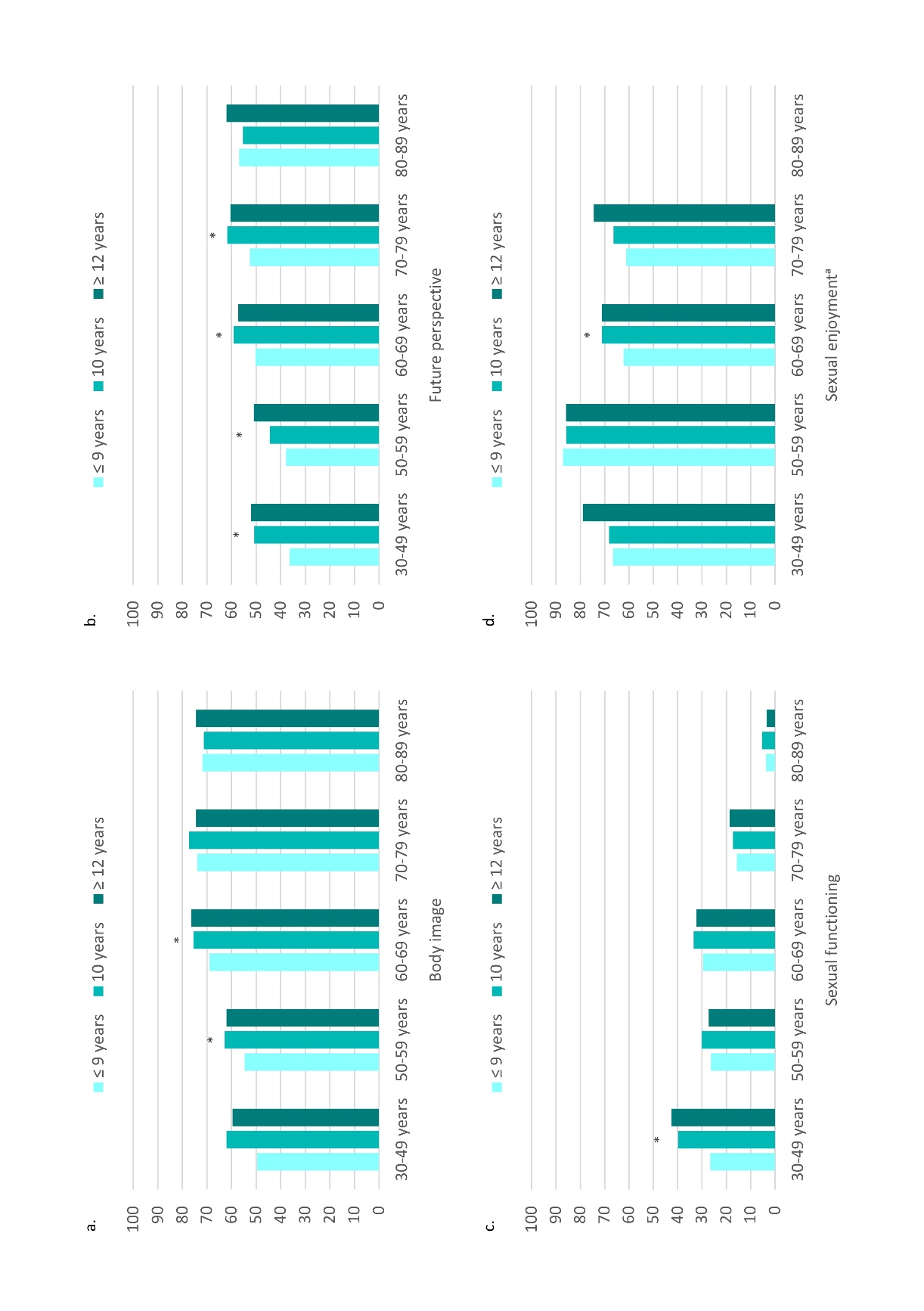


**Fig. S1:** EORTC BR23 functioning scores and items of long-term breast cancer survivors, stratified by age and education, adjusted for stage, type of surgery, chemotherapy, endocrine therapy, radiotherapy, lymph node dissection, time since diagnosis, and active disease.

*Footnotes: ^a^ conditional item (no imputation, no imputation, N = 934 participants). All further results are based on 25 imputations of missing values. The analysis is based on N = 2829 participants (exclusion of 216 BC survivors who either reported no surgery or an unusual combination of surgeries). High scores indicate better functioning. Asterisks (*) mark statistically significant differences in global comparison (p < .05).*


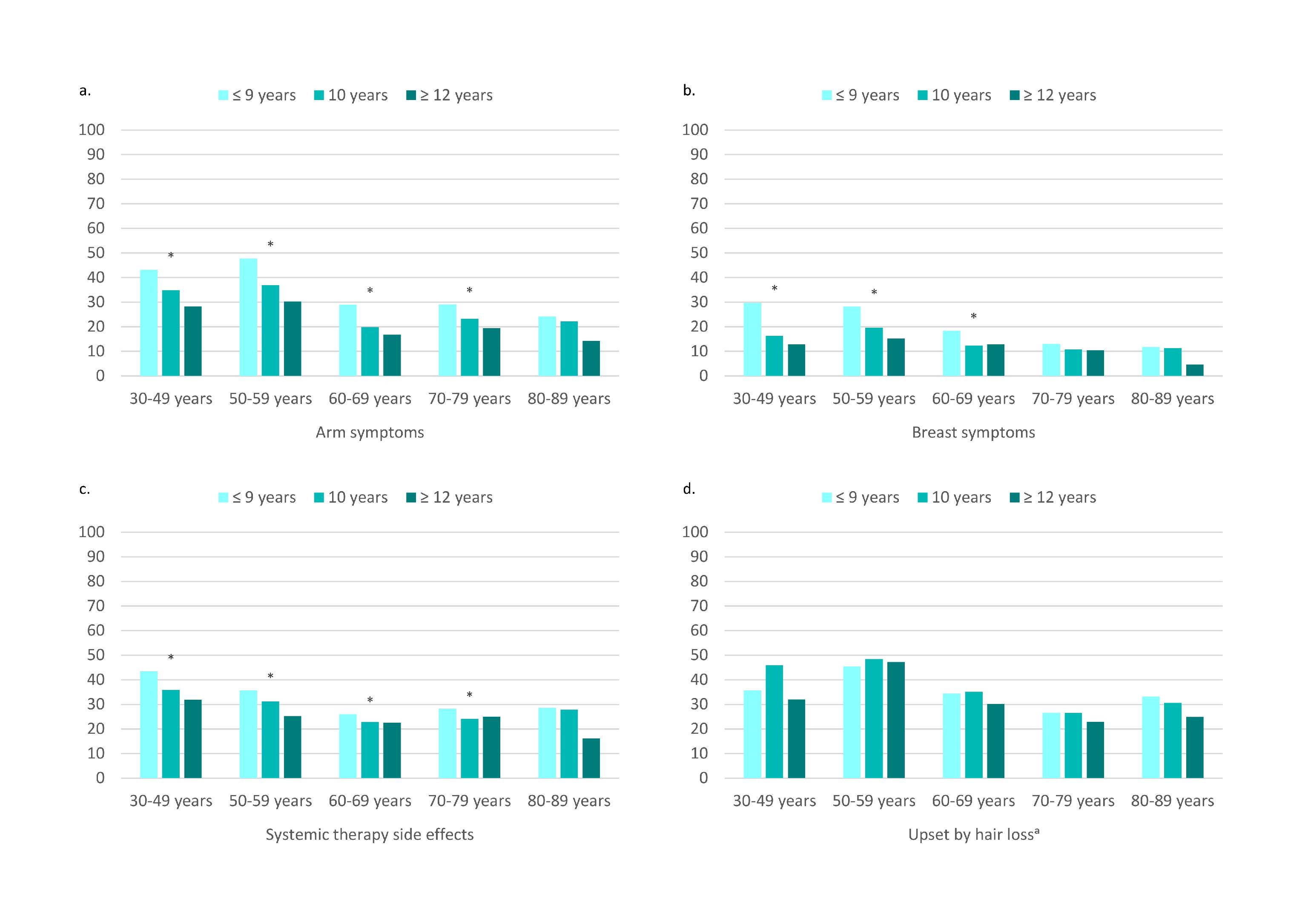


**Fig. S2:** EORTC BR23 symptom scores and items of long-term breast cancer survivors, stratified by age and education, adjusted for stage, type of surgery, chemotherapy, endocrine therapy, radiotherapy, lymph node dissection, time since diagnosis, and active disease.

*Footnotes: ^a^ conditional item (no imputation, N = 1442 participants). All further results are based on 25 imputations of missing values. High scores indicate greater burden. Asterisks (*) mark statistically significant differences in global comparison (p < .05).*
